# High dose fluconazole in salvage therapy for HIV-uninfected cryptococcal meningitis

**DOI:** 10.1186/s12879-018-3460-7

**Published:** 2018-12-12

**Authors:** Hua-Zhen Zhao, Rui-Ying Wang, Xuan Wang, Ying-Kui Jiang, Ling-Hong Zhou, Jia-Hui Cheng, Li-Ping Huang, Thomas Stephen Harrison, Li-Ping Zhu

**Affiliations:** 1 Department of Infectious Diseases, Huashan Hospital, Fudan University, 12 Central Urumqi Road, Shanghai, China; 2 Institute of Infection and Immunity, St George’s, University of London, London, SW17, 0RE UK

**Keywords:** High dose fluconazole, HIV-uninfected, Cryptococcal meningitis, Efficacy, Safety

## Abstract

**Background:**

The 2010 Infectious Diseases Society of America (IDSA) guidelines for management of cryptococcal diseases recommend high dose fluconazole (≥ 800 mg/day), either alone or with other antifungal drugs, as alternative anticryptococcal choices. But evidence for its use in the treatment of HIV-uninfected cryptococcal meningitis (CM) remains sparse.

**Methods:**

A retrospective analysis of HIV-uninfected CM patients who received fluconazole 800 mg/day for salvage therapy from January 2011 to December 2016 at Huashan Hospital, Shanghai, China was performed. Efficacy and safety were assessed, and mortality and prognostic factors evaluated.

**Results:**

A total of 44 patients were studied including 19 refractory to amphotericin B induction therapy, 8 refractory to fluconazole consolidation therapy (400 mg/d), and 17 intolerant of antifungal drugs. For salvage, 11 patients received triple therapy of high dose fluconazole, amphotericin B and flucytosine, 20 received dual therapy of high dose fluconazole and flucytosine, 13 received monotherapy of high dose fluconazole. Median duration of high dose fluconazole in salvage regimens was 136.5 days (range, 1–667 days). Clinical response rates were 72.1% (31/43) and 83.7% (36/43) when assessed at 2 weeks and the end of salvage therapy, respectively. Adverse events possibly related to high dose fluconazole occurred in 54.5% (24/44) of the patients, and all were mild or moderate. From the initiation of salvage therapy, 1-year all-cause mortality was 13.6% (6 of 44 patients) among the study population with no significant difference in refractory or intolerant patients.

**Conclusions:**

Adherence to guideline recommendations of high dose fluconazole, alone or in combination with other antifungals, was safe and often effective for salvage therapy of HIV-uninfected CM patients.

## Background

Cryptococcal meningitis (CM) is the most common opportunistic fungal infection of the central nervous system (CNS). The vast majority of cases are caused by *Cryptococcus neoformans*, while *Cryptococcus gattii* is more geographically restricted [1]. According to an updated estimate, over 220,000 cases of CM occur each year in HIV-infected patients, resulting in 181,000 deaths in 2014 [2]. Owing to the advent of highly active antiretroviral therapies, the incidence of HIV-infected CM has decreased and the long-term survival rate has increased in developed countries in recent years [3]. However, a growing number of CM cases occur in HIV-uninfected hosts with solid organ transplants, innate immunodeficiency and immune disorders, exogenous immunosuppressant administration, and even apparently normal immune status [4]. Mortality rate in these patients was as high as 25–40%, which was no lower than that in HIV-infected CM patients [5–8]. As a very heterogeneous group, HIV-uninfected CM patients pose great therapeutic challenges to clinicians.

Fluconazole is a triazole with excellent in vitro and in vivo activity against *Cryptococcus*. It has been widely used in CM as consolidation and maintenance therapy, usually at a dosage equal to or lower than 400 mg/day. In resource-limited areas, fluconazole alone has been used for induction therapy, and proved to be a poorly effective drug with 10-week mortality consistently > 50% even at high dosage [9–12]. But the efficacy was acceptable or favorable when high dose fluconazole was used in combination with other antifungal drugs or as enhanced consolidation therapy [13–18]. In the 2010 Infectious Diseases Society of America (IDSA) guidelines, higher dose fluconazole alone or in combination with other antifungals was recommended for both consolidation and salvage therapy in HIV-uninfected CM patients, compared with 400 mg/day in the 2000 version of these guidelines [19, 20]. However, evidence of high dose fluconazole for treatment of CM was mainly based on studies of HIV-infected populations, and remained sparse in HIV-uninfected patients. Therefore, we conducted a retrospective study among HIV-uninfected CM patients who received fluconazole 800 mg/day for salvage therapy, and evaluated the efficacy and safety of high dose fluconazole regimens.

## Methods

### Study design

This is a retrospective cohort study conducted among HIV-uninfected CM patients from January 2011 to December 2016 at Huashan Hospital, Fudan University (a tertiary health care center in Shanghai, China, with approximately 1200 hospital beds and 60,000 admissions per year). Patients who had been refractory to or intolerant of prior antifungal drugs and who then switched to salvage therapy with fluconazole 800 mg/day were included. This study was reviewed and approved by the local medical ethics committee.

### Definitions

A proven diagnosis of CM was made if the patient met any of the following criteria: (1) positive culture of *Cryptococcus* from cerebrospinal fluid (CSF), (2) positive CSF ink smear, (3) cryptococcal capsular polysaccharide antigen detected in CSF on cryptococcal antigen (CrAg) lateral flow assay (IMMY, Inc., Norman, Oklahoma, USA) in CSF or (4) compatible histopathological findings, which are 5–10 μm encapsulated yeasts in brain tissue. Refractory CM was considered if any 2 of the following conditions were present after an adequate period of antifungal therapy (≥14 days): (1) persistently positive cultures of *Cryptococcus* in CSF, (2) deterioration of clinical signs and symptoms of disease, (3) new sites of disease or worsening of pre-existing lesions radiologically, and (4) decreasing level of glucose and increasing level of protein in CSF [21, 22]. Patients were considered as intolerance to antifungal drugs if they suffered severe or life-threatening toxicity from amphotericin B (AmB) based initial therapy [22].

### Efficacy and safety assessment

Patients treated with high dose fluconazole for ≥1 week were included for efficacy evaluation. Response to salvage therapy was assessed at 2 weeks after initiation of salvage therapy, at the end of salvage therapy, and at the end of antifungal therapy. Efficacy of antifungal treatment was categorized as success (complete or partial response) or failure (stable response, disease progression, or death during the study period regardless of any cause) on the basis of clinical, radiological, and microbiological data according to previous criteria [21]. Adverse events (AEs) occurring during salvage therapy were recorded. The relationships of AEs and high dose fluconazole were evaluated with Naranjo probability scale [23]. High dose fluconazole related AEs were defined as one with a possible, probable or certain relationship.

### Statistical analysis

Continuous variables were compared with t test or the non-parametric Mann-Whitney test. Proportions were compared with the χ2 test or Fisher’s exact test, as appropriate. Log-rank test was used in univariate analysis for prognostic factors of 1-year survival, and Cox proportional hazards model for identification of independent prognostic predictors. A *P*-value of < 0 .05 was considered statistically significant.

## Results

### Demographics and manifestations

In total, 44 patients with proven CM were included in our study. Thirty-two also had pulmonary involvement and 6 cryptococcemia. The median age was 44 years, and 63.6% were male. Predisposing factors co-existed in 16 patients (36%). Detailed information is summarized in Table 1. Headache (100%) was the most common symptom, and 72.7% of them presented with fever, vomiting and meningeal irritation. Thirty-four patients presented with further neurological symptoms and signs, including altered mental status, epileptic seizures, cranial nerve deficits, limb weakness, uracratia and dysuria (Table 1).

**Table 1:** Baseline characteristics, signs and symptoms of 44 cryptococcal meningitis patients treated with high dose fluconazole for salvage therapy

Variables	No. (%) of patients
Sex, male	28 (63.6)
Age, years	44 (range, 16–73)
Predisposing factors	
Evans syndrome	4 (9.1)
MDS	1 (2.3)
ITP	1 (2.3)
Lymphoma	1 (2.3)
Solid tumor^a^	2 (4.5)
SLE	5 (11.4)
Cirrhosis	2 (4.5)
Liver transplantation	1 (2.3)
Splenectomy	1 (2.3)
Type 2 diabetes mellitus	2 (4.5)
Steroids or immunosuppressants	10 (22.7)
Headache	44 (100)
Fever	32 (72.7)
Vomiting	32 (72.7)
Meningeal irritation	32 (72.7)
Seizure	16 (36.4)
Altered mental status^b^	12 (27.3)
Cranial nerve defect	
Impaired vision	16 (36.4)
Diplopia	8 (18.2)
Visual field defect	8 (18.2)
Ophthalmodynia	3 (6.8)
Hearing impairment	18 (40.9)
Facial paralysis	3 (6.8)
Limb weakness	14 (31.8)
uracratia	3 (6.8)
Dysuria	3 (6.8)

*MDS* myelodysplastic syndrome; *SLE* systemic lupus erythematosus; *ITP* idiopathic thrombocytopenia

^a^Including 1 gastric cancer and 1 cholangiocellular carcinoma

^b^Patients with scores less than 15 by Glasgow Coma Scale

### Image findings

Cranial magnetic resonance imaging (MRI) was performed in all patients, with 41/44 (93.2%) yielding abnormal results. Local parenchymal lesions (86.4%) were most frequently observed, and common sites included frontal lobe (68.2%), parietal lobe (59.1%), periventricular region (29.5%) and basal ganglion (22.7%). Other abnormalities included meningeal enhancement (25 patients; 56.8%) and ventricular enlargement (12 patients; 27.3%). Most patients had 2 or more sites of involvement. Abnormalities on chest computer tomography (CT) scans were found in 32 of 44 patients (72.3%). The common findings were nodules and masses (47.7%), patches and strips (43.9%), pleural thickening (22.7%), pleural effusion (20.5%), lymph node enlargement (6.8%), air bronchogram sign (4.5%) and ground glass attenuation (2.3%). Some patients had mixed types of lesion on chest CT.

### CSF findings

Before initiating salvage therapy, CSF examinations were performed in 43 patients including 42 samples from lumbar punctures, 1 from continuous lumbar cerebrospinal fluid drainage. CSF cultures were positive for *Cryptococcus* in 4 patients, and ink smears were positive in 18 patients. CSF CrAg tests were positive in all 43 patients, and 47.7% of them (21/43) had CrAg titer of at least 1:640. Median white blood cell count (WBC) of CSF was 29 /mm^3^ (range, 0–550 /mm^3^). Median levels of CSF protein and glucose were 824 mg/dL and 34 mg/dL, respectively. Of the 42 patients who had lumbar punctures, 21 had CSF opening pressure above 25 cm H_2_O.

### Antifungal therapy

**Fig. 1:**
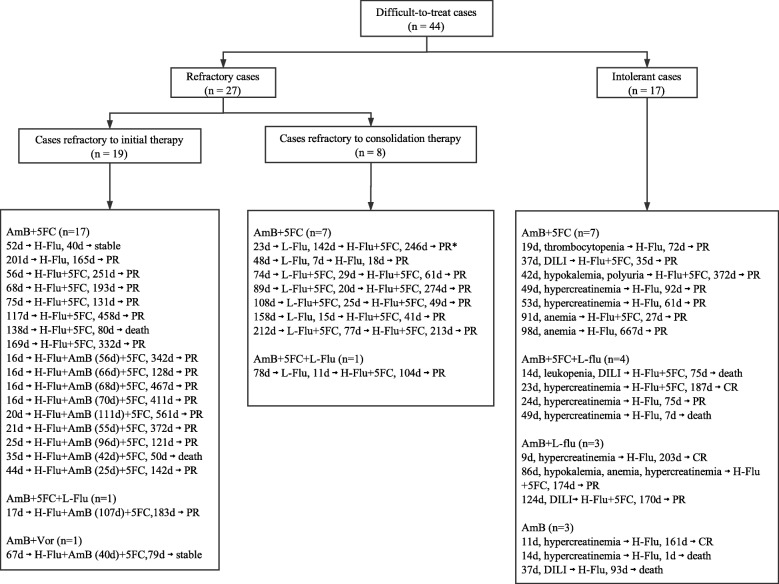
Profiles of initial therapy and salvage therapy in 44 difficult-to-treat cryptococcal meningitis patients. *AmB* amphotericin B; *5FC* flucytosine; *Vor* voriconazole; *L-Flu* low dose fluconazole (≤ 400 mg/day); *H-Flu* high dose fluconazole (800 mg/day); CR complete response; *PR* partial response; *DILI* drug induced liver injury. Addition or removal of 5FC during initial and salvage therapy were not listed. An example for reading the figure: * means that the patient received AmB and flucytosine for a duration of 23 days as induction therapy, followed by consolidation therapy with low dose fluconazole for 142 days, and then switched to salvage therapy with high dose fluconazole for 246 days, and finally achieved partial response at the end of salvage therapy

As listed in Fig. 1, 19 patients (43.2%) were refractory to initial antifungal therapy, 8 (18.2%) were refractory to consolidation therapy, and 17 (38.6%) were intolerant of prior antifungals. AmB-based regimens were initially administered in all 44 patients, and daily dosage of AmB ranged from 20 to 40 mg (0.4–0.7 mg/kg/day). Median cumulative dose and duration of AmB were 1205 mg (range, 205–5775 mg) and 48.5 days (range, 9–212 days).

Of the 19 patients refractory to initial therapy, 11 received high dose fluconazole in combination with AmB and flucytosine for salvage, 6 received high dose fluconazole and flucytosine, and the remaining 2 received high dose fluconazole alone. For patients who were refractory to consolidation therapy, fluconazole dosage was increased from 400 mg/day to 800 mg/day and flucytosine co-administered in all but one patient. Of the 17 antifungal-intolerant patients, 10 received high dose fluconazole alone, and 7 others were treated with high dose fluconazole and flucytosine. Overall, triple therapy of high dose fluconazole, AmB and flucytosine was administered in 11 patients, 20 received dual therapy of high dose fluconazole and flucytosine, 13 received monotherapy of high dose fluconazole. Median duration of high dose fluconazole in salvage therapy for the 44 patients was 136.5 days (range, 1–667 days). Among the 31 patients (70.5%) receiving flucytosine, 27 were treated with flucytosine throughout the course, and the remaining 4 discontinued the drug for suspected side effects (3 with anemia, 1 with elevated transaminase). Median duration of flucytosine in salvage therapy for the 31 patients was 142 days (range, 22–561 days).

### Efficacy and outcome

Except 1 patient who received high dose fluconazole less than 7 days and died of multiple organ failure (MOF), the other 43 patients were all included into efficacy evaluation. Two weeks after salvage therapy, partial response in 31 patients (72.1%), stable response in 11 patients (25.6%), and death caused by brain hernia in 1 patient (2.3%) were observed. At the end of salvage therapy, 3 patients (7.0%) achieved complete response, 33 (76.7%) achieved partial response, 2 (4.7%) achieved stable response, and 5 (11.6%) died. The overall efficacy rate was 83.7%. Of the 2 stable patients, 1 discontinued fluconazole, reinstituted with AmB and flucytosine and achieved partial response; the other was lost for follow-up 40 days after initiation of salvage therapy. Of the 4 patients who died after 2 weeks of salvage therapy, 1 was considered to have died of CM, 2 died from secondary infection and MOF, and 1 died from aggravation of lymphoma. Thirty-seven of 43 patients (86.0%) achieved clinical success and stopped antifungal drugs, 5 (11.6%) died and 1 (2.3%) was lost for follow-up. As shown in Table 2, we compared the response rates between patients treated with high dose fluconazole with or without flucytosie and those with combinations of AmB and high dose fluconazole with or without flucytosine. Although a significant higher 2-week response rate was observed in the triple combination group (62.5% vs. 100%, *P =* 0.019), there was no apparent differences in efficacy at the end of salvage therapy (84.4% vs. 81.8%, *P* >  0.999) and the end of antifungal therapy (84.4% vs. 90.9%, *P* >  0.999). At 1-year follow-up after stopping antifungal drugs, all of the 37 successfully treated patients survived, and no relapses were observed.

**Table 2:** Clinical success rate in 43 cryptococcal meningitis patients treated with high dose fluconazole for salvage therapy

Time point	Total (*N* = 43)	H-Flu ±5FC (*N* = 32)	H-Flu + AmB ± 5FC (*N* = 11)	*P-*value
2-week of Salvage Therapy	31 (72.1)	20 (62.5)	11 (100)	0.019
End of Salvage Therapy	36 (83.7)	27 (84.4)	9 (81.8)	> 0.999
End of Antifungal Therapy	37 (86.0)	27 (84.4)	10 (90.9)	> 0.999

*H-Flu* high dose fluconazole (800 mg/day); *AmB* Amphotericin B; *5FC* flucytosine

### Safety

All 44 patients were included in the safety assessment. Possible high-dose fluconazole related AEs were found in 54.5% (24/44) of them. No grade 3 or 4 AEs were observed. As shown in Table 3, the most frequent AE was elevated transaminase level in 11 patients. Transient grade 2 neutropenia and grade 1 thrombocytopenia occurred in 1 patient treated with fluconazole monotherapy. One patient presented with grade 2 anemia in the first week of salvage therapy, but his hemoglobin level recovered without discontinuance of fluconazole or flucytosine. Hypokalemia occurred in 3 patients, all of whom returned to normal condition after potassium supplementation (AmB were not co-administrated). There were no increased creatinine levels related to high dose fluconazole in our cases.

**Table 3:** High dose fluconazole related adverse events in 44 cryptococcal meningitis patients

Adverse events^*a*^	No. (%) of patients (*N* = 44)
Liver dysfunction	
GGT	10 (22.7)
ALT	5 (11.4)
AST	4 (9.1)
ALP	1 (2.3)
Hematocytopenia	
Anemia	1 (2.3)
Neutropenia	1 (2.3)
Thrombocytopenia	1 (2.3)
Hypokalemia	3 (6.8)
Nausea and vomitting	5 (11.4)
Constipation	3 (6.8)
Dry mouth	2 (4.5)
Dry eyes	1 (2.3)
Skin lesion	2 (4.5)
Numbness of upper limb	1 (2.3)
Thrombosis of lower limb	1 (2.3)

*GGT* gama-glutamyltransferase; *ALT* alanine aminotransferase; *AST* aspartate aminotransferase; *ALP* alkaline phosphatase

^a^All laboratory abnormalities were grade 1 or grade 2 only

In terms of clinical manifestations, gastrointestinal side effects were common complaints. Skin lesions were documented in 2 patients. Photosensitive dermatitis occurred in 1 patient soon after initiation of fluconazole, and improved without fluconazole discontinuance. Rash developed in another patient several weeks after the salvage therapy and lasted until CM recovered and antifungal treatment finished. We also observed 1 patient with elevated D-dimer and deep venous thrombosis which was relieved after treatment with oral warfarin. None of these patients discontinued high dose fluconazole due to intolerance.

### Mortality and prognostic factors

**Fig. 2:**
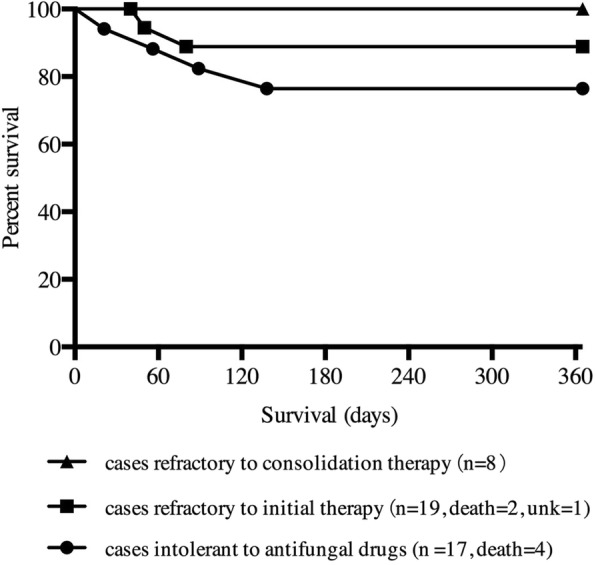
All-cause mortality in three groups of cryptococcal meningitis patients treated with high dose fluconazole for salvage therapy. *Unk* status unknown

From the beginning of salvage therapy, 1-year mortality related to all cause was 13.6% (6 of 44 patients) with no significant difference among 3 treatment groups (Fig. 2, *P* = 0.258). We assessed factors with a potential impact on all-cause mortality by means of univariate and multivariate analyses. In the univariate model, factors significantly associated with 1-year mortality were age ≥ 60 years (*P* = 0.001); serum albumin level < 35 g/L (*P* = 0.001); estimated glomerular filtration rate (eGFR) < 60 mL/min/1.73m^2^ (*P* = 0.015); and CSF WBC count < 10 /mm^3^ (*P* = 0.002). CSF CrAg titer ≥1280 was more frequently presented in mortality group but it did not reach a statistic significance (*P* = 0.058). By multivariate analysis, as shown in Table 4, factors significantly correlated with decreased survival in the overall population were serum albumin level < 35 g/L (*P* = 0.014) and CSF CrAg titer ≥1280 (*P* = 0.048).

**Table 4:** Univariate and multivariate analysis of factors associated with 1-year mortality among 44 cryptococcal meningitis patients

Factor	Univariate Analysis			Multivariate Analysis	
	Total No. of patients	No. (%) of patients who died	*P-*value^*b*^	*P-*value	RR (95% CI)
Age ≥ 60 years	12	4 (33.3)	0.022		
Initial AmB course < 6 weeks	10	3 (30.0)	0.095		
Salvage therapy without 5FC	13	3 (23.1)	0.196		
Serum albumin level < 35 g/L	13	5 (38.5)	0.001	0.014	16.23 (1.78–148.19)
eGFR < 60 mL/min/1.73m^2^	8	3 (37.5)	0.015		
CSF WBC count < 10 /mm^3a^	14	4 (28.6)	0.012		
CSF glucose level < 36 mg/dL^a^	22	1 (4.5)	0.127		
CSF CrAg titer ≥ 1280^a^	17	4 (23.5)	0.058	0.048	9.30 (1.02–84.73)

*AmB* Amphotericin B; *5FC* flucytosine; *eGFR* estimated glomerular filtration rate; *CrAg* cryptococcal capsular antigen; *CSF* cerebrospinal fluid; *WBC* white blood cell

^a^Data of CSF WBC count, CSF glucose level and CSF cryptococcal antigen titter was unavailable in 1 dead patient

^b^Factor entered the initial univariate analysis included male, age ≥ 60 years, time before diagnosis ≥60 days, predisposing factors, pulmonary involvement, seizure, altered mental status, parenchyma lesions of brain MRI, initial AmB course < 6 weeks, salvage therapy without 5FC, serum albumin level < 35 g/L, eGFR < 60 mL/min/1.73m^2^, CSF WBC count < 10 /mm^3^, CSF protein level > 500 mg/dL, CSF glucose level < 36 mg/dL, CSF CrAg titer ≥1280. Only predictors with a *P*-value less than 0.2 were listed in the table

## Discussion

In 1996, Pappas et al. reported 12 HIV-uninfected CM patients who received high dose fluconazole, 800 mg/day, in their induction therapy or as consolidation therapy [24]. However, since then, no published study has specifically addressed the use of high dose fluconazole in the management of HIV-uninfected cryptococcal infections. The results of our study showed that high dose fluconazole (800 mg/day) either alone or in combination with other antifungal drugs had favorable effects and was well tolerated among HIV-uninfected CM patients.

AmB-based regimens are recommended to optimize fungal clearance and survival for both HIV-infected and HIV-uninfected CM patients [19]. Despite given appropriate treatment, 25–35% patients experience refractory cryptococcal infections or drug intolerance [5, 6, 18, 25, 26]. In previous trials with AmB lipid complex, posaconazole and voriconazole used as salvage therapies, the overall efficacy rates were not impressive, with success rates no more than 50% [22, 27, 28]. Recombinant IFN-γ treatment as salvage therapy was tested only in case reports, and this form of adjunct could be detrimental in non-HIV infections, depending on host immune responses [29–31]. In our study, after salvage therapy with fluconazole 800 mg/day for a median of 137 days, 72% achieved effective response in 2 weeks, and 84% achieved effective response at the end of salvage therapy. Consistent with our findings, the good efficacy of high dose fluconazole as salvage therapy was also reported in HIV-infected CM patients, with 6 out of 8 patients (75%) achieving microbiological improvement after a mean course of 4.5 months, showed as either sterile CSF culture or decreased CSF CrAg titers [32]. Compared with patients treated with high dose fluconazole with or without flucytosine, there was significantly improved efficacy at 2 weeks in patients treated with AmB and high dose fluconazole with or without flucytosine. However, no apparent differences were showed in the long term outcomes between the two groups at the end of salvage therapy and the end of antifungal therapy.

In our study, no significant difference in 1-year mortality rates was found among patients with refractory infections and those intolerant of prior antifungal drugs. Factors linked with poor prognosis in our patients were older age, renal dysfunction, lower CSF white cell count, and high CSF CrAg titers, as consistent with previous reports in HIV-uninfected population [5, 6, 25, 33]. Though previous reports found altered mental status to be an additional factor, it was more common in patients died of active disease and not significant in our present study. Compared with an overall mortality rate of 25–40% reported in the previous studies of HIV-uninfected CM population [5–7], a relatively lower mortality rate of 13.6% after 1 year of salvage therapy was observed in our study.

In previous studies of treatment for cryptococcal or other fungal infections, fluconazole was well tolerated at dosages from 800 to 2000 mg/day [9–13, 34–37]. Consistent with previous studies, nausea and vomiting were common in patients treated with fluconazole, especially early in the course of treatment. Liver enzyme abnormalities were usually asymptomatic and self-limiting. In our study, 10 of the 17 antifungal-intolerant patients had AmB-related renal insufficiency and switched to salvage regimens. Of note, further deterioration of renal function related to high dose fluconazole was not observed, suggesting that high dose fluconazole with or without flucytosine were well tolerated for patients with AmB induced renal impairment.

There were some limitations to this work. As we included a limited number of cases in the present study and did not include a group of patients who received low dose fluconazole as a control, the results should be interpreted with caution. Only a few patients were culture positive when changed to salvage therapy. For these patients, the reasons for “failure” may be due to the poor efficacy of previous therapy, dysfunctional immune responses, or associated underlying conditions, and some may have done well if the previous antifungal therapy had been continued. Hence, multicenter randomized controlled trials are needed for making a further objective evaluation.

## Conclusions

Evidence for the use of high dose fluconazole in HIV-uninfected CM patients remains sparse. Our study specifically addressed the efficacy and safety of high dose fluconazole in this population and suggested that fluconazole 800 mg/day alone, or in combination with other antifungal drugs was a safe and promising choice for HIV-uninfected CM patients. Either refractory cases or those intolerant to prior antifungal drugs can achieve favorable outcomes after receiving high dose fluconazole as salvage therapy.

## Abbreviations

AEs: Adverse events
AmB: Amphotericin B
CM: Cryptococcal meningitis
CNS: Central nervous system
CrAg: Cryptococcal antigen
CSF: Cerebrospinal fluid
CT: Computerized tomography
HIV: Human immunodeficiency virus
IDSA: Infectious Diseases Society of America
MOF: Multiple organ failure
MRI: Cranial magnetic resonance imaging
WBC: White blood cell
